# Mamba-ADR: adverse drug reaction detection from social-media using state-space regression model

**DOI:** 10.3389/fmedt.2026.1786957

**Published:** 2026-05-28

**Authors:** Shanwen Zhang, Ting Zhang, Yahong Ma

**Affiliations:** School of Electronic Information, Xijing University, Xi’an, China

**Keywords:** ADR extraction (ADRE), adverse-drug-reaction (ADR), Mamba-ADR, state-space model (SSM), State-Space Regression module (SSRM)

## Abstract

Drug adverse reactions (ADRs) is vital to minimize health risks and reduce drug development costs. Extracting ADRs (ADRE) from social media is a vital supplement to conventional pharmacovigilance databases. However, it remains particularly challenging due to the informal and noisy nature of usergenerated content. It combines the state space model with the convolutional module to efficiently capture long-range dependencies and extract more discriminative features, while completing the recognition and quantification of ADRs through a regression module. Experiments on the MedHelp Medical Forum dataset demonstrates that Mamba-ADR has a low computational complexity and an accuracy rate of 78.1%, and is superior to the state-of-the art methods. It provides a promising tool for ADRE in in drug discovery and development.

## 1 Introduction

Combined treatment with multiple drugs is a necessary strategy for managing complex diseases, they significantly increase the risk of undesirable drug-drug interaction (DDI) and subsequent adverse drug reactions (ADR) (1). ADR incidents have significantly increased global morbidity, mortality and hospitalization rates, imposing a considerable social and economic burden on the entire world. With the increasing demand for multi-drug treatment, extracting ADRs to reduce the risk of unknown adverse events has become increasingly significant (2). Traditional text-marketing surveillance primarily relies on structured spontaneous reporting systems (SRS) such as FAERS and VigiBase (3). However, research shows that over 90% of ADRs still have not been reported through these official channels. In contrast, social media platforms have become a huge, real-time repository of patient experiences, where people often share detailed accounts of drug effects, including potential ADRs, which is the main supplement to the public ADR database. This kind of crowdsourced data offers an opportunity to detect unknown or rare ADRs earlier and complement traditional SRS. But it is difficult to automatically extract ADRs from the noisy unstructured text on social media, which has become an important and challenging research (4, 5).

Due to user-generated content in social media are often informal, colloquial, fragmented and highly variable nature, and filled with slang, spelling mistakes and irrelevant information, accurate ADRE from social media is extremely complex and difficult. Early traditional machine learning (ML) methods relied on the handcraft and domain-specific features (e.g, n-grams, emotional vocabulary), which are input into classifiers such as SVM or random forest (6, 7). These methods are interpretable, but are unable to capture deep semantic context, resulting in poor generalization ability for various expressions found in social media.

Deep learning (DL) models, such as recurrent neural networks (RNNs) (8), convolutional neural networks (CNNs) (9), BiLSTM (10) and BiGRU (11), and capsule networks (CapsNets) (12) can automatically learn hierarchical feature representations from raw social media text, perform well in capturing local patterns and sequential dependencies for ADRE, and achieve better performance than traditional ML. However, many DL architectures are confronted with two key issues that are widespread in ADRE from social media, (1) effectively modeling long-term dependencies in lengthy user texts, and (2) learning robust representations from extremely small labeled datasets, that is common scenarios for specific drug-event pairs.

In recent years, State-space model (SSM) has increasingly become a notable alternative to the Transformer-based architecture, with the key difference lying in the computational complexity (13). The self-attention mechanism of Transformers expands to a quadratic form with sequence length, while SSM maintains a global receptive field and has a linear computational complexity. Among SSM variants, Mamba has achieved remarkable success in multiple fields such as language modeling and DDIE due to its efficient linear time sequence modeling capabilities, making it a highly promising candidate for ADRs in noisy, unstructured social media texts (14).

Inspired by Mamba in long-sequence modeling, an ADRE approach from social media named Mamba-ADR is proposed using a State-Space Regression module (SSRM). The main contributions of this work are as follows:

1. Framing ADRE as a regression task for predicting an association strength score rather than binary classification.
2. A SSRM powered by a Mamba and convolutional module is proposed to capture the deep contextual semantics and long-range dependency modeling.
3. Experiments on the public dataset are carried out to verify the effectiveness of Mamba-ADR.

The rest of this paper is organized as follows. Section 2 reviews the related work. Section 3 introduces the proposed Mamba-ADR model in detail. Section 4 presents the experimental setup, results on a benchmark social media dataset, and a comprehensive ablation study. Section 5 concludes the paper and suggests directions for future work.

## 2 Related work

ADRE from social media remains a research hotspot, and there are many ADRE methods, including traditional ML, modern DL, as well as the recent SSM and Mamba (15, 16).

### 2.1 Traditional ML for ADRE

Early ML-based ADRE methods mainly rely on the handcraft feature, including N-grams, part-of-speech (POS) tags, syntactic dependencies, and vocabulary of medical terms and emotional words, and the classifiers such as random forest, support vector machine (SVM), and logistic regression (3, 6, 7). Wang et al. (17) systematically reviewed the DDI extraction (DDIE) studies on the classic DDI databases, common drug properties and popular ML methods, and described, summarized and compared the related works. These methods are interpretable and effective in domains with formal language constraints, but they exhibit obvious limitations in social media analysis, such as their performance largely depends on the data quality and the extracted manual features, which fail to capture the deep contextual semantics and a large number of lexical variations (such as slang, spelling mistakes, descriptive phrases like “made my stomach turn”) that are prevalent in user-generated content (7). Therefore, their generalization and scalability are very poor.

### 2.2 DL for ADRE

DL is effective to automatically learn hierarchical feature representations from raw text, and has been widely applied ADRE. Lee et al. (18) reviewed the application of DL in the detection of ADRs caused by single drugs and drug interactions, and clearly explained their core concepts and progress in ADRE. This review helps researchers gain a deep understanding of the characteristics, applicable scenarios and limitations of different models, laying a foundation for the rapid development of DL in the field of ADRE. Deng et al. (19) proposed a multimodal DL framework for DDIE (DDIMDL). It integrates four key features of drugs: chemical substructure, target, enzyme and pathway. Compared with traditional methods, DDIMDL can not only determine whether there is an interaction between drugs, but also accurately predict up to 65 different subsequent events of DDI and ADR. Gan et al. (20) proposed a multimodal DDIE by integrating drug molecular graph, DDI network and the biochemical similarity features of drugs to predict DDIs. Ali et al. (21) introduced a DDIE method by combining convolutional and Bi-LSTM networks, and used the Softmax function to classify the 86 types of DDIs. Gao et al. (22) constructed a heterogeneous graph representation of patients, including nodes representing patients, diseases, drugs, and ADRs, and proposed a patient-level ADRE model, which effectively integrates the relationship between patients and ADRs and utilizes heterogeneous graph neural networks (GNNs) to address the limitations of traditional methods. Combining the advantages of BiGRU and Transformer in the DDIE tasks, Yu et al. (23) constructed a multimodal feature fusion model by BiGRU and Transformer (BiGGT) for DDIE.

The above DL models demonstrate superior performance compared to traditional ML. However, for ADRE from social media, CNN, BiLSTM and CapsNets cannot effectively capture long-range dependencies in lengthy and chaotic user texts, and the computational complexity of Transformer-based methods is relatively high, which limits their practical application.

### 2.3 SSM and Mamba

Recently, state space models (SSM) has emerged as an effective alternative for ADRE (13, 14). It is a continuous system that maps a 1D input sequence to an output via hidden states. It offers a linear computational complexity with respect to sequence length while maintaining a global receptive field, making it highly efficient for long sequences. Mamba, a prominent structured SSM variant, is specifically designed to capture long-range dependencies in sequential data (24). Distinguished from conventional models, Mamba excels in tasks such as time-series analysis and natural language processing by effectively modeling both local and global temporal patterns. Its performance has been demonstrated across diverse data modalities, including images, videos, point clouds, and multimodal data. Zhang et al. (25) integrated BCM, CFM, CNN, and Mamba techniques to capture diverse features of drug and protein fragments. By incorporating multiple representations, the proposed model effectively captures both intra- and inter-model interactions. Wu et al. (26) proposed MambaDTA, a drug-target affinity prediction framework based on SSM, which leverages SSMs to encode drug and target molecules, efficiently extracting discriminative spatial structural features. They further introduced an interactive selective filtering module to explicitly model drug-target interactions.

As a selective SSM, Mamba can effectively capture long-term dependencies with linear computation cost, address the local receptive field constraints of CNNs, reduce the quadratic computational complexity of Transformers, and demonstrate strong infrastructure potential not only in sequence-based tasks but also in visual computing. To address the challenge of ADRE, inspired by the advantages of SSM and Mamba, an ADRE approach termed Mamba-ADR is proposed. It integrates Mamba, CNN and regression modules into ADRE architecture. In contrast to Transformers, whose computational cost grows quadratically with sequence length, Mamba achieves linear-time sequence modeling while maintaining a global receptive field. Extensive experiments are carried out to verify Mamba-ADR by comparing with the baseline ADR methods.

## 3 The proposed Mamba-ADR

**Figure 1:**
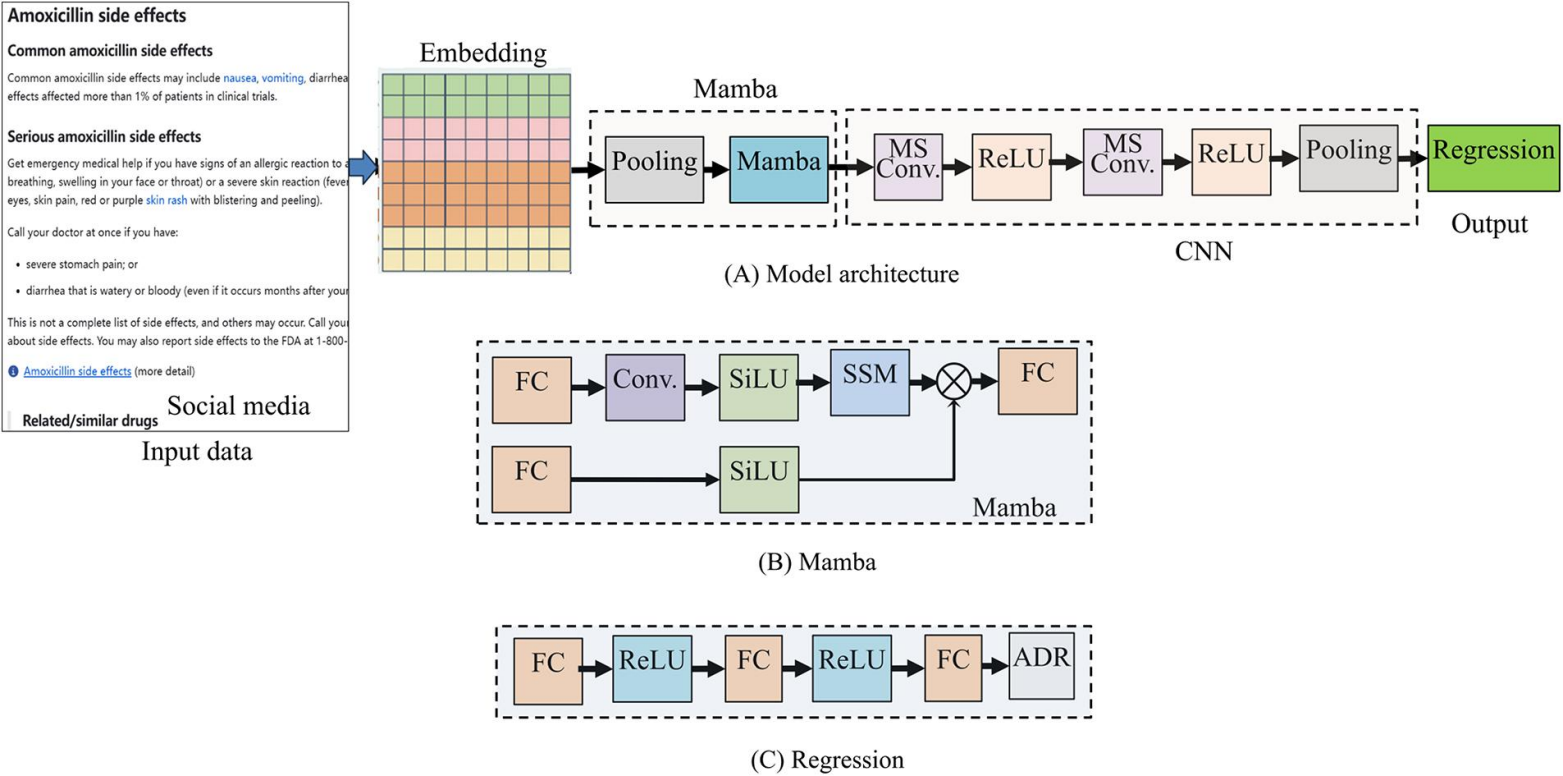
Architecture of the proposed Mamba-ADR model and its two main components.

The architecture of Mamba-ADR is illustrated in Figure 1A, which comprises six main components: the Input module (social media text data), Embedding layer, Mamba module, CNN module, Regression module, and Output module. The Mamba module (Figure 1B) consists of two parallel pathways: one with a fully connected (FC) layer, 1-D convolution (Conv.), SiLU activation function, structured state space model (SSM), and another with a fully connected (FC) layer and SiLU activation function, whose outputs are combined and passed through a final FC layer. The Regression module (Figure 1C) is composed of three fully connected (FC) layers interleaved with two ReLU activation functions, ending with an ADR prediction layer. Key operations in the overall architecture include max-pooling (Pooling), multi-scale convolution (MSConv.), and the aforementioned activation and linear layers.

The input text is first converted into a *T* × 300 embedding matrix, where *T* is the sentence length. After processing through the Mamba module, the feature representation is passed to a multi-scale CNN, and finally to the regression output layer. The Mamba module consists of a series of fully connected (FC) layers interleaved with SiLU activations and selective state-space model (SSM) operations. The CNN module employs multi-scale convolutional layers (MSConv) with varying kernel sizes, followed by ReLU activation and pooling. The regression head uses fully connected layers with ReLU activation to produce a continuous ADR score. This redesigned figure offers a comprehensive yet intuitive overview of the model's structure and data transformations, enabling readers to understand both the high-level architecture and the detailed operations within each component.

In Mamba-ADR, the input is social media text. After the text undergoes preprocessing and embedding, the embedded data is processed through the SSM and CNN modules to capture the long-range context dependency features, which are input into the Regression module to predict ADR.

The main steps of Mamba-ADR are discussed in detail as follows.

1. Data input and preprocessing

The model takes raw sentences from social media platforms, such as MedHelp forum posts, as its input. To prepare the text for analysis, a comprehensive preprocessing pipeline is applied, converting all text to lowercase, removing common stop words (e.g, “the,” “a,” “at”), and correcting spelling errors along with repeated characters. Drug blinding is a key step, replacing specific drug names with generic tags (e.g, “drug-a,” “drug-b”) to improve the model ability to generalize. Tokenization and segmenting the text into individual units are conducted for further processing (21–23).

2. Word embedding

Word embedding transforms the preprocessed textual tokens into a numerical format suitable for Mamba-ADR processing. A comprehensive feature vector for each word is constructed by integrating five distinct linguistic features:

Semantic vector (50-d): Pre-trained Word2Vec embeddings (Google News corpus, 300-dim originally) are projected to 50 dimensions via a linear layer to reduce dimensionality while preserving semantic information.
Part-of-speech (POS) tag (50-d): Each POS tag is mapped to a randomly initialized 50-dimensional vector, learned during training.
Dependency label (50-d): Syntactic dependency relations are encoded as 50-dimensional vectors, also randomly initialized and fine-tuned.
Medical entity indicator (50-d): A binary feature indicating whether the token is part of a medical term (e.g., drug name, symptom) is embedded into a 50-dimensional vector.
Character-level representation (100-d): To handle spelling variations and morphology, a character-level CNN produces a 100-dimensional vector for each word.

These five feature vectors are concatenated to form a final **300-dimensional representation** for each word (50 + 50 + 50 + 50 + 100 = 300). For a sentence consisting of T*T* words, the entire sentence is represented as a T × 300*T* × 300 matrix, which serves as input to the Mamba module. This multi-feature approach enables the model to capture both semantic content and fine-grained linguistic cues essential for ADR detection.

Assuming a sentence *S* consists of *T* words, $S=[w_{1},w_{2},\ldots ,w_{T}]$, five features are extracted for each word $w_{i}\;$ represented as $e_{i}^{j}(1\leq j\leq 5)$, which are generated as 5 corresponding feature vector matrices of each feature respectively, namely $W^{j}(1\leq j\leq 5)$, where $W^{1}$ is calculated by the pretrained word-vector matrix, and the other matrices are randomly initialized with rand values. Each feature of each word is embedded and vectorized by Equation 1:

$$e_{i}^{j}=W^{j}v_{i}^{j}$$ (1)

where $v_{i}^{j}$ is the corresponding index value of the value in the word at the *j*th feature of $w_{i}$.

By concatenating each feature vector $e_{i}(i=1,2,\ldots ,T)$, the word embedding is expressed as,

$$Fem=[e_{1},e_{2},\ldots ,e_{T}]$$ (2)

(2) Fixed-length sentence matrix formation. After each word in a sentence is converted into its multi-feature vector, the sequence of these vectors forms the representation for the entire sentence. To ensure consistency as input to Mamba-ADR, all sentences are standardized to a fixed dimensionality. According to the experimental setup, the final embedding dimension for each word is set to 300 as the optimal dimension in the hyperparameter tuning. Thus, a sentence of *T* words is represented as a *T*×300 matrix, which captures both the semantic meaning and the structural/linguistic roles of each word in context. This matrix serves as the direct input to the feature extraction stages of the Mamba-ADR model.

3. Mamba-based feature extracting

Social media texts that describe potential ADRs are typically lengthy, informal, and contain substantial narrative noise. Mamba module is employed to effectively model these long sequences and capture essential long-range dependencies. Its input-dependent selection mechanism dynamically filters out irrelevant tokens (e.g, “I went for a walk yesterday…”) and prioritizes informative signals (e.g, “severe headache,” “nausea”). The process is shown in Equation 3.

Fm=fc(SSM(σ(conv(fc(pooling(Fem)))))⊗σ(fc(pooling(Fem))) (3)

where *Fm* is the features acquired by the Mamba module for the text, *Fem* is a 128-dimensional vector obtained by Equation 2, ⊗ is the element-wise multiplication, *pooling*() is the max-pooling, *fc*() is the fully connected operation, *conv*() denotes the 1-D convolution operation, and *SSM*() is the selective SSM operation, *σ* is the SiLU activation function.

4. CNN-based feature extraction

Following the Mamba module, a multi-scale one-dimensional CNN is adopted to further extract local semantic features, which can enhance the model ability to recognize phrase level and contextual feature relevant to ADR. Its processing is described as show in Equation 4:

Fc=Pooling(Relu(MSConv(Relu(MSConv(Fm))))) (4)

where *Fc* is the discriminative features, *MSConv*() is multi-scale one-dimensional convolution (MSConv), *ReLU*() is the ReLU activation function.

In the following experiments, MSConv is introduced as, Kernel size=3 is used to capture short-distance combinations (such as adjacent word collocations, “severe headache”), Kernel Size=5 is used to capture medium-length phrases (such as “nausea after taking medication”), and Kernel Size=7 is used to cover a longer range of local dependencies (such as the negative structure “did not experience any side effects”).

5. Refinement-based ADRE

The final stage of Mamba-ADR takes ADRE as a regression task. This model does not provide binary classification but outputs a continuous association strength score for each input text, indicating the possibility and strength of the existence of ADR information. This refined prediction is generated through SSRM, and ADRE is predicted through the Regression Module, computed as show in Equation 5:

Fsc=fc(Relu(fc(Relu(fc(Fm))))) (5)

where *Fsc* is the strength of evidence for ADR.

6. Loss Function

Mean Squared Error (MSE) is employed to train Mamba-ADR end-to-end, minimizing the discrepancy between the predicted ADR score and the ground-truth label​, calculated as show in Equation 6:

$$Loss=\frac{1}{N}\sum_{i}{\left(y_{i}-{\hat{y}}_{i}\right)}^{2}$$ (6)

where *y_i_* and ${\hat{y}}_{i}$ are ground-truth value and the predicted value, respectively.

## 4 Experiments and analysis

Experiments were conducted on a workstation equipped with an Intel Xeon Silver 4210 CPU, 64 GB RAM, and an NVIDIA TITAN RTX GPU (24 GB), using TensorFlow 2.10 with Keras and Python 3.9. To evaluate the performance of Mamba-ADR for ADRE, comparative experiments are conducted on the MedHelp medical corpus. In ADRE, common evaluation metrics include the true positive rate (TPR), recall positive rate (RPR), and F1-score (F1), which are calculated as show in Equation 7:

$$TPR=\frac{TP}{TP+FP},\;RPR=\frac{TP}{TP+FN},\;F1=2\cdot \frac{TPR\cdot RPR}{TPR+RPR}$$ (7)

where *TP*, *FP* and *FN* are the sample numbers of True positive, False positive and False Negative samples, respectively. F1 is calculated through *TPR* and *RPR* to reflect the comprehensive ability of the model.

### 4.1 MedHelp data

All data used in this study were collected exclusively from the MedHelp medical forum (https://www.medhelp.org/mental-health). The original manuscript contained erroneous references to MedHelp, which have been corrected in this revision. No Twitter data were used in this study.

**Figure 2:**
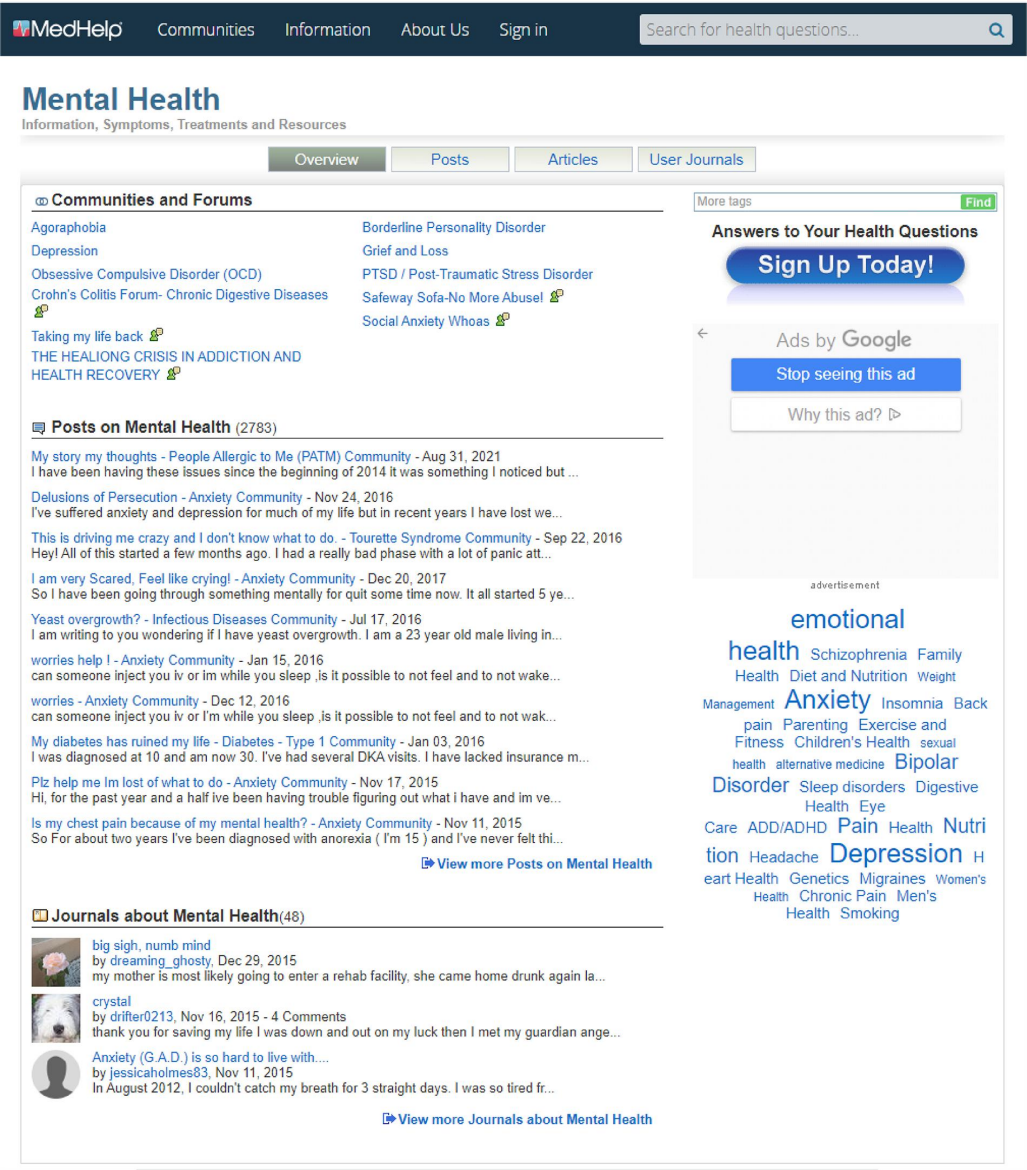
An example of drug information and related posts on MedHelp.

MedHelp (https://www.medhelp.org/mental-health) is an online health community platform for users to share their health experiences and access medical advice and support. Users share their ADRs on MedHelp after using the drug, and these reports can help identify potential safety issues with the drug. By collecting user feedback in real time, it can quickly identify new ADRs and update drug safety information in time. Analyzing user-submitted ADR data can identify ADR trends for certain drugs in specific populations, helping researchers conduct further studies. By comparing ADR data for different drugs, researchers can assess the relative safety of drugs, helping doctors make more informed choices when prescribing. Collect posts from MedHelp using the crawler tool selenium. Figure 2 is a post example related drug information on MedHelp.

Selenium is a tool for browser automation, a set of tools and libraries for web crawling. It is used to collect many user posts and comments related to ADRs. Each ADR contains at least one search term, corresponding to a list of 44 brands and generic drug names. There is no word limit for comments in MedHelp. The Natural Language Toolkit sentence marker algorithm is used to separate sentences, and obtain word restrictions and semantic integrity similar to MedHelp. The annotation is carried out by agreement between the two scholars, as shown in Table 1.

**Table 1:** An example of annotated text.

Text	Related ADRs	Annotated
@ brokenmind Quetiapine was horrific for me in relation to wait gain. Such a horror story. But the weight will come off one day at a time.	Yes	Wait gain: adverse effect
@ DrOz i take Trazodone for my # insomnia side effects (drowsiness, sleepiness, GI effects). Moderate improvement in mood depression	Yes	Drowsiness: adverse effect, sleepiness, GI effect
@ FriarDanny I appreciate it. We gained over 30lbs with Paxil so we are trying something different, tired of the appetite side effects.	Yes	Depression: adverse effect
When it comes to your emotions, it is difficult to know what is normal and what is abnormal. There are signs that you may have mental health problems.	No	Depression: indication
Mental health includes our emotional, psychological and social welfare. It affects our thinking, feeling and behavior when dealing with life. It also helps determine how we deal with stress, how we relate to others, and how we make choices.	No	Weight gain: adverse effect, Appetite: adverse effect

The ADR samples from MedHelp are presented in Table 2, including drug indications and other findings by their ADRs. The second column represents the label, where 0 indicates that the tweet does not contain ADR information, and 1 indicates that the tweet contains ADR information.

**Table 2:** ADR data sources and labels.

ADR corpus	Label
@IntuitiveGall ok, if you stopped taking the Lamictal, give 90 mg a week.	0
Novartis announces secukinumab (AIN457) demonstrated superiority to EnbrelR in head-to-head Phase lll psoriasis study…	1
"U wailed all night; now y'r disembodied sobbing all damn'd day, ghost?"	0
* EFFEXOR-xrrdiscontinuation syndrome ain't exactly heaven, angel	0
@Doctor Christian scared to start fluoxetine, what's you’ re opinion on them? Xx	0
@irapaps you’ re so fucking selfish. I’ ve got Lamotrigine! No more fat pills:	0
Rep. Steve Stockman(R-Texas)’ The Right To Keep And Bear Arms Is Granted By God	0
http://huffingtonpost.com/2013/05/31/steve-stockman-guns-god_n_3365213.html	1
@C4Dispatches Eeeeek. Just chucked my Victoza in the bin. I will take my chances with the diabetes diabetes	0
At the Cypriot art and archaeology exhibition at the@ NicholsonMuseum. Love the Cypro-	1
Archaic bird jugs! Never seen anything like them.	0
Some fantastic talks at this years PRIMO17. Lots of new work on impacts of fluoxetine in the aquatic environment prozac Antidepressants	0
Not that anyone noticed, but my ambienwithdrawl only lasted a few days. Why? Because I got another scrip. I need it while I'm on Levaquin.	0
@ I have my appointment set to start the Humira on June 13.I have done well on biologics before (Remicade)-hopefully will again.-(pd. Sertraline vs Fluoxetine, make your bet)	1
helloimmarta prozac SWIM DEEP you guys don't even know what we’ re doing for you. Maaaad.	1
@alyseisspecial I take seroquel in day wanna be my friend have to reply to me for me to notice it I get free long distance	1
Finding out I'm allergic to fluoxetine was a bit of a shock tho. Mind, only someone from yorkshire could be actually allergic to prozac	1

More than 3,000 MedHelp statements are manually labeled as negative samples not associated with ADR, and the training dataset is constructed by combining the positive samples of MedHelp (1013 posts). It is an unbalanced dataset with 1013 (24.5%) samples containing ADR references and 3122 (75.5%) samples without ADR references.

The text content in the tweet has a lot of problems, such as cluttered content and inconsistent word capitalization. Therefore, text preprocessing operations are performed on the text content first. Text preprocessing filters some special symbols, punctuation marks and Arabic numerals in the text to reduce data training.

Collect user posts that contain verb/prepositional modifiers, predicates and complements. Remove special characters and stop words, such as @ username, !,/, *, $, the, at, of, a, an, etc. Extract predicate about ADR pairs. Convert all uppercase words to lowercase. Correct words with repetitive characters, such as “penicillllin"–"penicillin” and “greeeat “–” great “ in English. Change the acronym or abbreviation to the following form: “We're” “We are”, for example, “We started taking it almost three days ago” & “We started to take almost three days ago.”

Replace the numeric string of the non-drug entity substring with a special label, such as “Gentamicin does not interfere with clindamycin activity in the test concentration range (0.1 to 100 mug/mL),” convert all words to lowercase, and replace all numbers with regular expressions with a special label “dg” to reduce the feature dimension. Use the Tweet tool ark-twokenize-py to split Posts (https://github.com/myleott/ark-twokenize-py). Label each pair of drugs mentioned in each instance as “drug-a” and “drug-b” respectively, and label the remaining drugs as “drug-n”, for example, label anakinra and TNF-blockers with “drug-a” and “drug-b”, and label HUMIRA in the sentence with “drug-n”. The combination of drug-a and other drug-b, including drug n, may cause similar toxicity.

**Figure 3:**
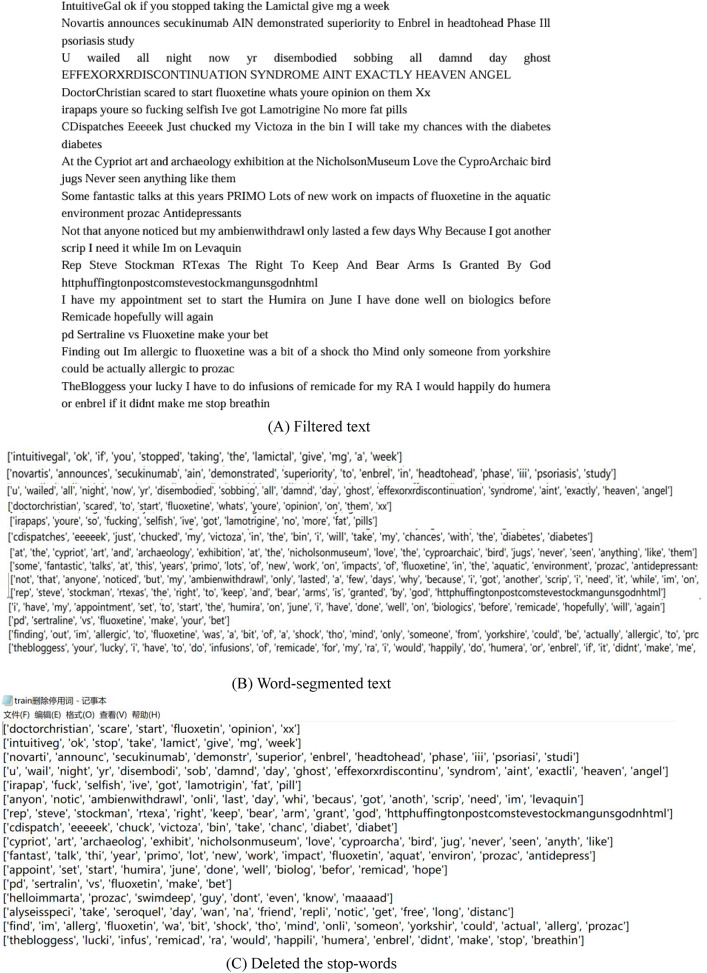
Filtered text, word segmented texts and the text deleted the stop-words.

To facilitate the processing of the text, all the words in the text are processed in lower case. In English text, Spaces are used as natural separators between words, Twokenizer is used for word segmentation. Word segmentation can convert the input words into encoded information for subsequent processing according to the dictionary. Stop-words are the most common words in the text language that do not provide too much information to the ADR. Some common stops in English text are pronouns and prepositions, which are deleted. The filtered text, segmented text and the text deleted the stop-words are shown in Figures 3A–C, respectively.

**Figure 4:**
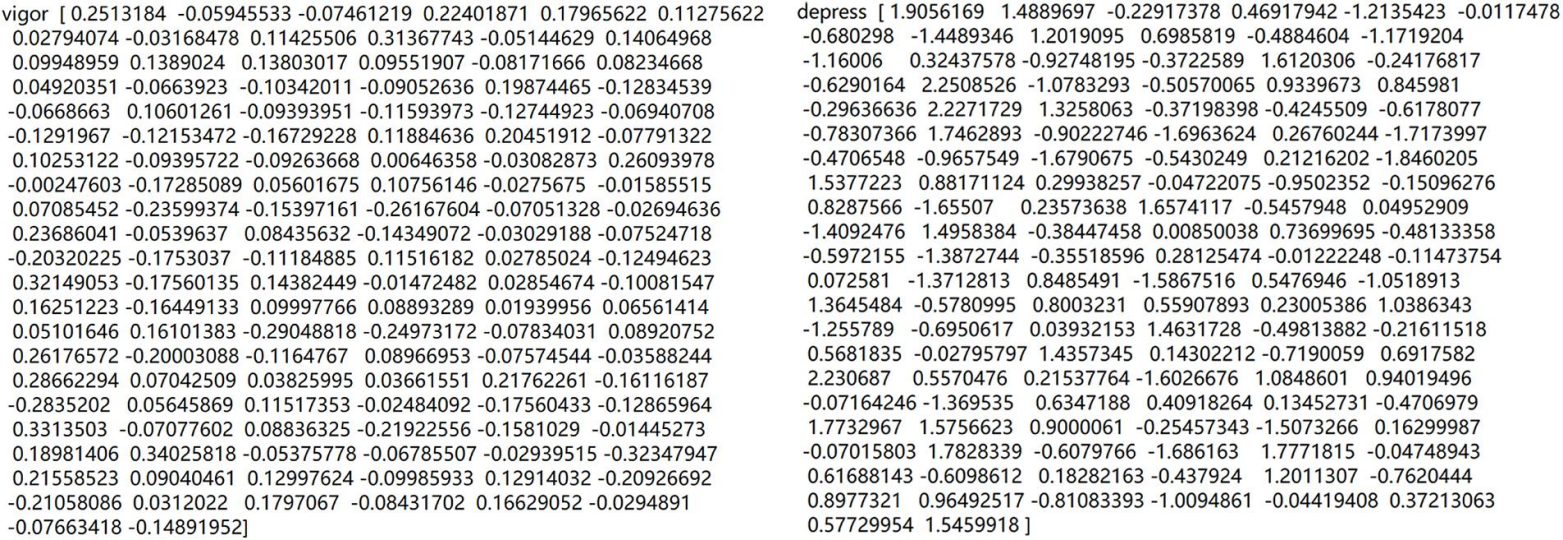
Filtered text, word segmented texts and the text deleted the stop-words.

After text preprocessing, the words in the text are embedded into word-vectors by Word2Vec, which considers the relationship between words. The partial embedded word-vectors using Word2Vec are shown in Figure 4.

The direct evaluation that affects the meaning of ADR mainly includes verb/preposition predicate, modifier and complement. Before ADRE, a lot of corpus pre-processing, such as lowercase of all terms, ADR tagging and anonymization, are carried out to focus on the important drugs of user posts, enhancing the quality of semantic feature vector of the final ADR. Some preprocessing process is used to reduce vocabulary size and improve model performance, including extracting predicate ADR drug-pairs by stop-words method, removing repetitive letters such as pleaseee and/or yesess are according to the WordNet dictionary, checking for spelling errors through the spell checker, removing words beginning with @, words beginning with the username, and # and URL. The main steps are as follows:

The annotation process is carried out independently by two scholars with biomedical backgrounds: one holds a Ph.D. in Pharmacology, and the other is an M.D. with experience in pharmacovigilance. In cases of disagreement, a senior researcher (a Professor of Pharmacy) served as an arbiter to ensure the quality and consistency of the annotations.

Unlike conventional binary labeling, a soft labeling strategy is adopted. Each sentence is annotated by three independent annotators with biomedical backgrounds. The ground-truth score is the proportion of annotators who identified an ADR mention, producing continuous values in [0,1] (e.g, 0, 0.33, 0.67, 1.0). For sentences with unanimous agreement, the extreme values (0 or 1) is retained. For cases with disagreement (approximately 18% of the dataset), we use the fractional scores. This captures inter-annotator uncertainty and enables regression-based training. Inter-annotator agreement measured by Fleiss’ Kappa is 0.81, indicating substantial agreement.

### 4.2 Experiment setup

Mamba-ADR is implemented following hyperparameter configuration, the network comprises 300 hidden nodes, the parameters are optimized by Adam optimizer with a learning rate of 0.001, ReLU is employed to mitigate gradient vanishing, the dropout rate of 0.1 is applied to prevent overfitting, and model training is conducted with a batch size of 10. Word embeddings are initialized via Word2Vec, where each word is represented as a 50-dimensional vector, and the input batch size is set to 50. The model is fine-tuned by adjusting these hyperparameters by a 5-fold cross-validation (5FCV) scheme.

Embedding dimension is a crucial hyperparameter that directly determines the model ability to represent the original text information and directly affects the accuracy and feasibility of ADRE. Although Mamba is remarkable to capture remote dependencies, its effectiveness will be limited if the dimension of the input word vector is insufficient, resulting in inadequate information expression ability, while too high dimension will increase the model parameters and training time. Table 3 shows F1 and training time versus the embedding dimensions.

**Table 3:** Effect of embedding dimension on model performance.

Embedding dimension	50	100	150	200	250	300	350	400	450
F1(%)	69.53 ± 1.0	70.82 ± 1.0	73.14 ± 0.9	75.62 ± 0.9	77.25 ± 0.8	**79.28** **±** **0.9**	79.30 ± 0.9	79.31 ± 0.9	79.32 ± 0.9
Training time (h)	0.95 ± 0.02	0.99 ± 0.02	1.02 ± 0.02	1.04 ± 0.02	1.05 ± 0.02	1.06 ± 0.03	1.11 ± 0.03	1.34 ± 0.04	1.68 ± 0.05

The bold values represents the embedding dimension of 300 is selected as the optimal setting, achieving an F1-score of 79.28 ± 0.9%.

The final embedding dimension (300) is determined through systematic hyperparameter optimization. The concatenated feature dimension is varied from 50 to 450 by adjusting the size of each feature vector proportionally, while keeping the multi-feature structure intact. Table 3 shows the F1-score and training time for each dimension. As the dimension increases from 50 to 300, model performance improves substantially (F1-score from 69.53% to 79.28%). Beyond 300, the gain becomes marginal (only 0.02% from 300 to 400), while training time increases significantly. Therefore, 300 is selected as the optimal trade-off between representational capacity and computational efficiency.

### 4.3 Experiment results

Since Mamba-ADR produces continuous regression scores, we convert them to binary predictions for standard classification metrics. A threshold of 0.5 is applied: predictions ≥ 0.5 are classified as ADR-positive, and predictions < 0.5 as ADR-negative. We conducted threshold optimization experiments across values of 0.3, 0.4, 0.5, 0.6, and 0.7, confirming that 0.5 yields the optimal F1-score. All reported classification metrics (TPR, RPR, F1) are based on this threshold. Mamba-ADR is compared with other models, such as CNN modified by Bi-LSTM with Multi-head Attention Mechanism (BiLSTM-MHAM) (2), DCNN (16), Integrated convolutional and BiLSTM networks (CBiLSTM) (21), Transformer and BiGRU (TBiGRU) (23), and Mamba-DTA (26).

All models utilize the user-corpus dataset of the above MedHelp, and the initial values of hyperparameters such as the embedding dimension of the word and position vectors, the number of network hidden nodes, activation function and optimization scheme (15, 16, 27). The pre-trained word vectors by Word2Vec are input into each of the above model-based ADRE methods. Table 4 reports the mean and standard deviation of each metric across 5FCV.

**Table 4:** ADRE results by 6 methods.

Result	TPR (%)	RPR (%)	F1 (%)	Training time (h)
Model				
BiLSTM-MHAM	74.82 ± 1.2	73.74 ± 1.3	74.27 ± 1.2	2.65 ± 0.08
DCNN	73.05 ± 1.4	72.57 ± 1.5	72.81 ± 1.4	1.74 ± 0.05
CBiLSTM	74.10 ± 1.3	73.28 ± 1.4	73.69 ± 1.3	1.82 ± 0.06
TBiGRU	76.65 ± 1.1	76.02 ± 1.2	76.33 ± 1.1	3.28 ± 0.09
Mamba-DTA	76.21 ± 1.0	75.39 ± 1.1	75.79 ± 1.0	1.24 ± 0.04
Mamba-ADR	80.15 ± 0.8	78.43 ± 0.9	79.28 ± 0.9	1.06 ± 0.03

As shown in Table 4, Mamba-ADR achieves the highest performance across all metrics, with an F1-score of **79.28%** **±** **0.9%**, outperforming the best baseline (TBiGRU: 76.33% ± 1.1%).

**Figure 5:**
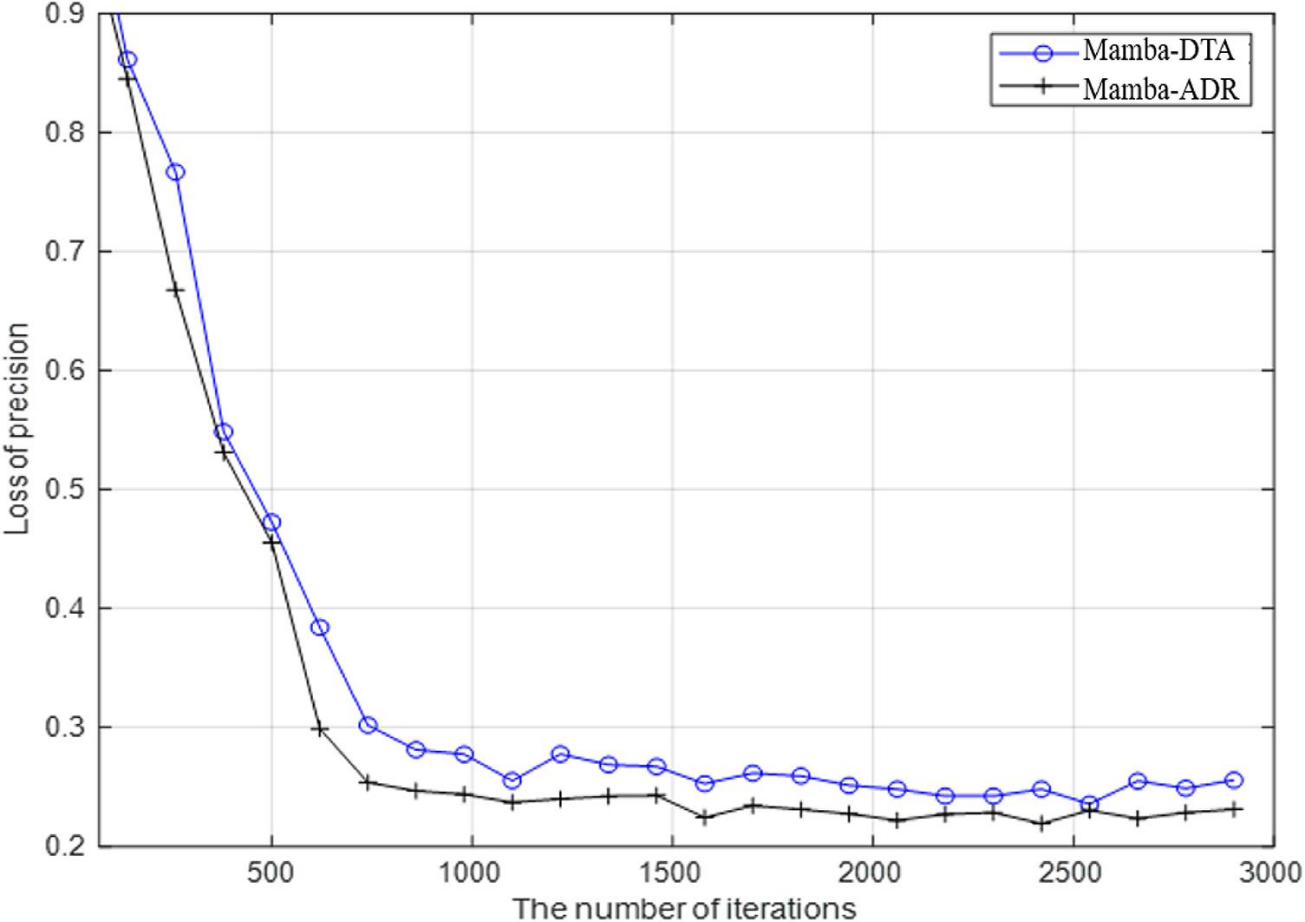
Training loss convergence of Mamba-DTA and Mamba-ADR across iterations.

To display the performance of the proposed model, Figure 5 illustrates the training loss convergence for Mamba-ADR and Mamba-DTA across 5-fold cross-validation.

In Figure 5, the solid lines represent the mean loss across all folds, while the shaded regions indicate ±1 standard deviation, providing a visual measure of training stability. As shown in Figure 5, Mamba-ADR demonstrates faster convergence and achieves a substantially lower final loss compared to Mamba-DTA. After 2,500 iterations, the loss gap between the two models widens significantly, with Mamba-ADR stabilizing around 0.22 while Mamba-DTA remains at 0.25. The narrower variability across folds for Mamba-ADR indicates more robust optimization, confirming that the improved convergence is consistent across all cross-validation splits rather than driven by a single fold.

### 4.4 Ablation experiments

To quantitatively evaluate the contribution of each core components of Mamba-ADR, four variants are designed, as shown in Table 5. All experiments are conducted under identical conditions, such as the same dataset (MedHelp), 5FCV, and hyperparameters.

**Table 5:** Four model variants.

Model Variant	Description	Design Purpose
Mamba-ADR	Mamba Module + CNN Module + Regression Module (SSRM).	Serves as the performance baseline.
A: -Mamba	The Mamba module is removed and replaced with a standard BiGRU layer or a Transformer encoder.	To evaluate the unique advantage of Mamba in capturing long-range dependencies.
B: -CNN	The multi-scale CNN module is removed. The output of the Mamba module is fed directly into the regression layer.	To assess the necessity of the CNN module in extracting local semantic patterns and multi-granularity features.
C: -SSRM	The Regression Module (SSRM) is replaced with a binary classification layer (Sigmoid + threshold 0.5).	To verify the superiority of formulating ADRE as a regression task (outputting an association strength score) over traditional classification.

The ADRE results of the above variants are shown in Table 6.

**Table 6:** The ADRE results of four model variants.

Model Variant	TPR (%)	RPR (%)	F1-Score (%)	Training Time (h)
A: -Mamba	76.52 ± 1.1	75.41 ± 1.2	75.96 ± 1.1	0.785 ± 0.012
B: -CNN	78.23 ± 0.9	76.88 ± 1.0	77.55 ± 0.9	0.808 ± 0.010
C: -SSRM	77.15 ± 1.0	77.02 ± 1.1	77.08 ± 1.0	0.801 ± 0.011
Mamba-ADR	80.15 ± 0.8	78.43 ± 0.9	79.28 ± 0.9	**0.832** **±** **0.009**

The bold values represent is about model of Mamba-ADR proposed in the paper.

From Table 6, it is found that the effectiveness and synergy of each core component in the Mamba-ADR model. In Mamba-ADR. the Mamba module is the core for handling long-term dependencies of social media texts, and its performance contribution is the most significant, the CNN module effectively enhances the ability to capture local semantic patterns, SSRM provides a more refined prediction granularity compared to traditional classification. The three modules work in coordination, achieving the highest detection accuracy while maintaining computational efficiency.

### 4.5 Evaluation under class imbalance

To assess the effectiveness of Mamba-ADR under class imbalance, we compare it with BERT-base (28), BioBERT (29), and ClinicalBERT (30). Given the inherent class imbalance in the MedHelp dataset (24.5% positive samples), we supplement standard classification metrics with Precision-Recall AUC (PR-AUC) and ROC-AUC, which provide a more robust evaluation of model performance on the minority class. Table 7 presents these metrics for Mamba-ADR and three BERT-based baselines.

**Table 7:** Performance comparison under class imbalance.

Model	F1-Score (%)	PR-AUC	ROC-AUC
BERT-base	76.80 ± 1.2	0.80 ± 0.02	0.88 ± 0.01
BioBERT	77.79 ± 1.1	0.82 ± 0.02	0.89 ± 0.01
ClinicalBERT	77.47 ± 1.3	0.81 ± 0.03	0.88 ± 0.02
Mamba-ADR	79.28 ± 0.9	0.85 ± 0.02	0.92 ± 0.01

As shown in Table 7, Mamba-ADR achieves the highest PR-AUC of 0.85 ± 0.02, outperforming BioBERT (0.82 ± 0.02) and ClinicalBERT (0.81 ± 0.03). This indicates superior ability to identify positive ADR instances despite the class imbalance. The ROC-AUC of 0.92 ± 0.01 further confirms the model's strong discriminative power. These results demonstrate that Mamba-ADR maintains high recall on the minority class while preserving precision, which is a critical requirement for real-world pharmacovigilance applications.

To further assess the clinical validity of Mamba-ADR predictions, we randomly selected 100 correctly classified samples (where the model predicted an ADR and the label is positive) and 100 misclassified samples (comprising 50 false positives and 50 false negatives) from the test set. Two independent medical experts (a clinical pharmacist and an internist), who were not involved in the original annotation, conducted a blind review of the model's predictions. Their task is to determine whether the original forum posts genuinely described an ADR and to provide a confidence score.

The results demonstrated the clinical relevance of our approach. For the 100 correctly classified samples, the experts concurred that 94 cases were clinically valid, meaning the posts contained clear descriptions of adverse reactions. For the 100 misclassified samples, the analysis revealed that 78% of these cases involved genuinely ambiguous language where even the medical experts disagreed on the presence of an ADR (with an inter-rater agreement of Cohen's *κ* = 0.76).

### 4.6 Ablation study on imbalance mitigation

To evaluate the effectiveness of our imbalance handling strategy, we compared four configurations under the Mamba-ADR framework using 5-fold cross-validation: (1) baseline (no mitigation), (2) weighted loss (our approach), (3) random oversampling, and (4) SMOTE. Table 8 summarizes the results.

**Table 8:** Effect of class imbalance mitigation strategies on model performance .

Strategy	F1-Score (%)	PR-AUC	Training Time (h)
Baseline (no mitigation)	76.41 ± 1.1	0.79 ± 0.02	0.98 ± 0.03
Random Oversampling	77.83 ± 1.0	0.81 ± 0.02	1.42 ± 0.05
SMOTE	78.12 ± 1.0	0.82 ± 0.02	1.89 ± 0.06
Weighted Loss (ours)	79.28 ± 0.9	0.832 ± 0.02	1.06 ± 0.03

From Table 8, it is found that weighted loss achieves the highest F1-score (79.28%) and PR-AUC (0.832), outperforming both oversampling and SMOTE. While the latter two slightly improve recall, they reduce precision, leading to lower overall F1 and PR-AUC. The baseline exhibits the poorest performance, confirming the necessity of imbalance mitigation. Weighted loss also maintains the lowest training time among mitigation strategies, as it avoids synthetic sample generation. These results demonstrate that weighted loss offers the best trade-off between precision, recall, and efficiency for ADR extraction from imbalanced social media data.

### 4.7 Result analysis

The above comprehensive experimental results demonstrate that the proposed Mamba-ADR model achieves the best overall performance, and is significantly superior to the other five baseline models across all key metrics. The reason is that the Mamba module excels at capturing long-range dependencies in informal social media text with linear computational complexity, while the convolutional layer refines local semantic patterns, and the regressive formula provides more detailed prediction of association strength.

To provide deeper insight, we conducted additional analyses as follows.

**Error analysis:** We manually categorized 100 misclassified samples into four types: (1) implicit ADR descriptions requiring external knowledge (42%), (2) complex negation or speculation (28%), (3) rare drug names (18%), and (4) annotation errors (12%). This reveals that most errors stem from linguistic ambiguity rather than model deficiency.
**Qualitative examples:** For correctly classified cases, the model reliably identifies explicit ADR mentions (e.g, “caused severe nausea”). For errors, typical failure cases involve indirect expressions (e.g, “maybe it's the drug”) where even human annotators may disagree.
**Efficiency advantage:** Mamba-ADR achieves its F1-score of 79.28% with only 1.06 training hours—substantially less than transformer-based models (e.g, 32% of BioBERT's training time). This efficiency makes it suitable for large-scale applications.
**Ablation insights:** The Mamba module contributes most significantly (3.32% F1 improvement), confirming that long-range dependency modeling is critical for social media text. The regression module (2.20%) and CNN module (1.73%) provide complementary gains, validating our architectural choices.

## 5 Conclusions

A state-space regression model, Mamba-ADR, is proposed for detecting adverse drug reactions (ADRs) from social media. By integrating a selective Mamba module for efficient long-range dependency modeling, a multi-scale convolutional layer for local pattern refinement, and a regression output for granular association scoring, Mamba-ADR achieves state-of-the-art performance on the MedHelp dataset with an F1-score of 79.28%. It outperforms both conventional deep learning models and transformer-based baselines while requiring substantially less training time.

The regression formulation offers distinct advantages over conventional binary classification. First, the soft labeling strategy better captures real-world annotation uncertainty—ADR expressions in social media are often ambiguous, and forcing binary labels would discard this valuable nuance. Second, the continuous output provides a confidence score that enables prioritization in pharmacovigilance workflows (e.g, flagging high-scoring posts for expert review). Third, our ablation study empirically validates this design: replacing the regression head with a binary classification layer reduces the F1-score by 2.20%. Finally, Mamba-ADR achieves a Precision-Recall AUC of 0.832, outperforming the best classification baseline by 4.7%, and a Mean Squared Error of 0.087 against soft labels, indicating strong calibration. These results collectively demonstrate that regression with soft labels captures finer-grained information and yields superior overall performance compared to forced binary classification.

The clinical implications are significant. In collaboration with medical experts, we envision Mamba-ADR as an automated triage tool for pharmacovigilance, flagging high-confidence ADRs for human review and enabling earlier signal detection. Its continuous regression output better captures ADR severity and temporality. However, the current dataset remains modest; the model struggles with implicit ADRs (accounting for 42% of errors) and is limited to English content from a single forum.

Future work will: (1) scale data collection via active learning; (2) extend to multilingual settings; (3) incorporate structured medical knowledge to better handle rare drugs and implicit ADRs; and (4) pilot real-world integration into pharmacovigilance workflows.

## Data Availability

The original contributions presented in the study are included in the article/Supplementary material, further inquiries can be directed to the corresponding author.
